# Induction of G2/M Phase Arrest by Diosgenin via Activation of Chk1 Kinase and Cdc25C Regulatory Pathways to Promote Apoptosis in Human Breast Cancer Cells

**DOI:** 10.3390/ijms21010172

**Published:** 2019-12-25

**Authors:** Wen-Ling Liao, Jing-Yi Lin, Jia-Ching Shieh, Hsiao-Fong Yeh, Yi-Hsien Hsieh, Yu-Chun Cheng, Huei-Jane Lee, Chen-Yang Shen, Chun-Wen Cheng

**Affiliations:** 1 Graduate Institute of Integrated Medicine, China Medical University, Taichung 40202, Taiwan; wl0129@gmail.com; 2 Center for Personalized Medicine, China Medical University Hospital, Taichung 40202, Taiwan; 3 Institute of Biochemistry, Microbiology and Immunology, Chung Shan Medical University, Taichung 40201, Taiwan; windy87518@hotmail.com (J.-Y.L.); maple@hotmail.com (H.-F.Y.); hyhsien@csmu.edu.tw (Y.-H.H.); lhj@csmu.edu.tw (H.-J.L.); 4 Department of Biomedical Sciences, Chung Shan Medical University, Taichung 40201, Taiwan; jcs@csmu.edu.tw; 5 School of Medicine, Fu Jen Catholic University, Taipei 24205, Taiwan; yuchuncheng0817@gmail.com; 6 Institute of Biomedical Sciences, Academia Sinica, Taipei 11529, Taiwan; 7 Graduate Institute of Environmental Science, China Medical University, Taichung 40202, Taiwan; 8 Clinical Laboratory, Chung Shan Medical University Hospital, Taichung 40201, Taiwan

**Keywords:** diosgenin, breast cancer, cell cycle, Chk1, apoptosis, ∆Ψm

## Abstract

The anti-tumor activity of diosgenin, a new steroidal constituent present in fenugreek, on two human breast cancer cell lines, MCF-7 and Hs578T, was studied. Diosgenin treatment resulted in cell growth inhibition, cell cycle arrest, and apoptosis in concentration- and time-dependent manners in both cell lines. Western blot analyses of whole cell lysates for cell cycle proteins showed that diosgenin altered phosphorylated cyclin checkpoint1 (p-Chk1^Ser345^) and cyclin B expression, which resulted in G2/M phase blockade. Mechanistically, Cdc25C-Cdc2 signaling was involved in inactivating Chk1^Ser345^ by p53-dependence in MCF-7 cells and p21-dependence in Hs578T cells that are p53-deficient. Moreover, diosgenin induced a significant loss of the mitochondrial membrane potential in breast cancer cells, and prominently affected cell death through down-regulation of the anti-apoptotic protein, Bcl-2. This released cytochrome c and activated the caspase signaling cascade. Taken together, these findings reveal that the anti-proliferative activity of diosgenin involves the induction of G2/M phase arrest via modulating the Cdc25C-Cdc2-cyclin B pathway and mitochondria-mediated apoptosis in human breast cancer cell lines. This suggests the potential usefulness of diosgenin in treating breast cancer.

## 1. Introduction

Breast cancer is the most common malignancy among women and the second leading cause of cancer-related death in the United States (http://seer.cancer.gov). Similar to western populations, an increasing female breast cancer incidence is being observed in Taiwan, and breast cancer has been the fourth leading cause of cancer-related death in women in Taiwan since 2006 [1]. A clear tendency of both an increasing incidence rate and number of victims has been evident over the past two decades. Thus, therapeutic advances with cost-effective strategies in reducing mortality caused by breast cancer are highly desirable.

Intensive research into the discovery of natural products has focused on the anti-tumor properties of phytochemicals to develop new cancer intervention strategies. A number of anticancer drugs, including camptothecin [2,3], docetaxel [4,5], licochalcone [6,7], polyphyllin I [8], and dioscin [9], are of plant origin. Recently, chemoprevention has been examined both in vitro and in vivo, and chemopreventive agents derived via phytomedicine have been validated in human intervention trials [10].

Diosgenin ([25R]-5α-spirosten-3β-ol), an aglycone, is a natural steroidal sapogenin found in a variety of plants (e.g., it can be extracted from fenugreek seeds and the root of the wild yam, *Dioscorea villosa*) that is used as a starting material for more than 60% of the industrial steroidal compounds. Diosgenin has a unique structural similarity to estrogen. In addition to functioning as a lactation aid, this bioactive substance is known for its medicinal qualities, such as antidiabetic and hypocholesterolemic effects, and can be used to treat non-alcoholic fatty liver disease [11]. It also has antioxidant and immunomodulatory properties [11,12,13,14]. Current research on diosgenin has shown that it possesses anticancer properties by inducing differentiation of human erythroleukemia cells through changes in lipoxygenase activities [15], promoting cell cycle arrest [16], and targeting several molecules involved in inhibiting cell proliferation, invasion, migration, angiogenesis, and metastasis of various types of human cancer [16,17,18,19].

However, to our knowledge, the ability of diosgenin to inhibit breast cancer cells has not been fully explored, and there is no evidence that the anticancer capability of diosgenin shown in vitro will affect the cell cycle checkpoint kinases (Chks) that control tumorigenesis. To this end, we determined the anti-proliferative activity of diosgenin by using two human breast cancer cell lines, MCF-7 and Hs578T, as in vitro models. In addition, to examine the effects of diosgenin on the cell cycle and apoptotic pathways, we determined its mechanism by examining cell cycle regulatory proteins (Chk1, Chk2, p21, Cdc25C, Cdc2, and cell cyclins) with respect to p53-responsiveness (MCF-7 cells) and p53-non-responsiveness (Hs578T cells). The results provided the first information on the ability of diosgenin to inhibit various breast cancers.

## 2. Results

### 2.1. Diosgenin Inhibits Breast Cancer Cell Proliferation

**Figure 1:**
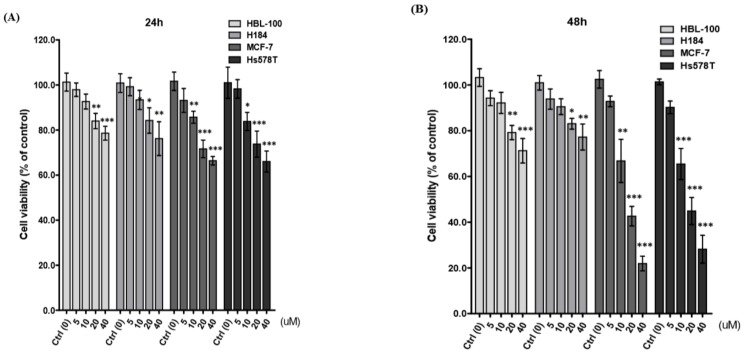
Effect of diosgenin on the growth of breast cancer cells. Human breast cancer MCF-7 and Hs578T cells, and normal mammary epithelial HBL-100 and H184B5F5/M10 cells, were incubated with the indicated concentrations of diosgenin for (**A**) 24 and (**B**) 48 h. Growth inhibition of cells by diosgenin was assessed by the MTT assay. Results are presented as percentages of viable diosgenin-treated cells relative to control cells (Ctrl, 0 µM). Data are shown as means ± SD from four independent experiments (each in triplicate). The viability of control cells is defined as 100%. * *p* < 0.05, ** *p* < 0.01, and *** *p* < 0.001 vs. control (Ctrl) cells.

Initially, we determined the growth inhibitory activity of diosgenin on two breast cancer cell lines, MCF-7 and Hs578T, and two non-malignant mammary epithelial cell lines, HBL-100 and H184B5F5/M10, by treating them with increasing concentrations of diosgenin (0–40 µM) for 24 and 48 h. The MTT assay showed that diosgenin significantly inhibited cell viability in concentration- and time-dependent manners (Figure 1A,B). Incubation with 40 µM diosgenin for 48 h resulted in an inhibition of approximately 80% (78.10 ± 3.53) for MCF-7 and 70% (71.8 ± 6.10) for Hs578T cells, as compared to that of HBL-100 and H184B5F5/M10. These findings indicated that both MCF-7 and Hs578T cells were sensitive to diosgenin treatments; thus, we selected these two breast cancer cell lines to perform further analyses and evaluate the anti-tumor property of diosgenin.

### 2.2. Diosgenin Induces G2/M Arrest in Breast Cancer Cell Lines

**Figure 2:**
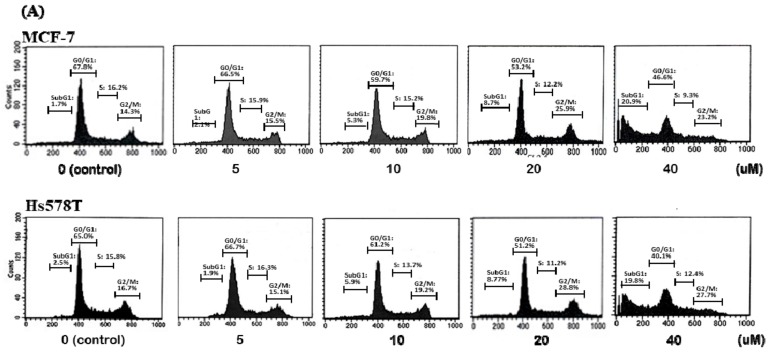

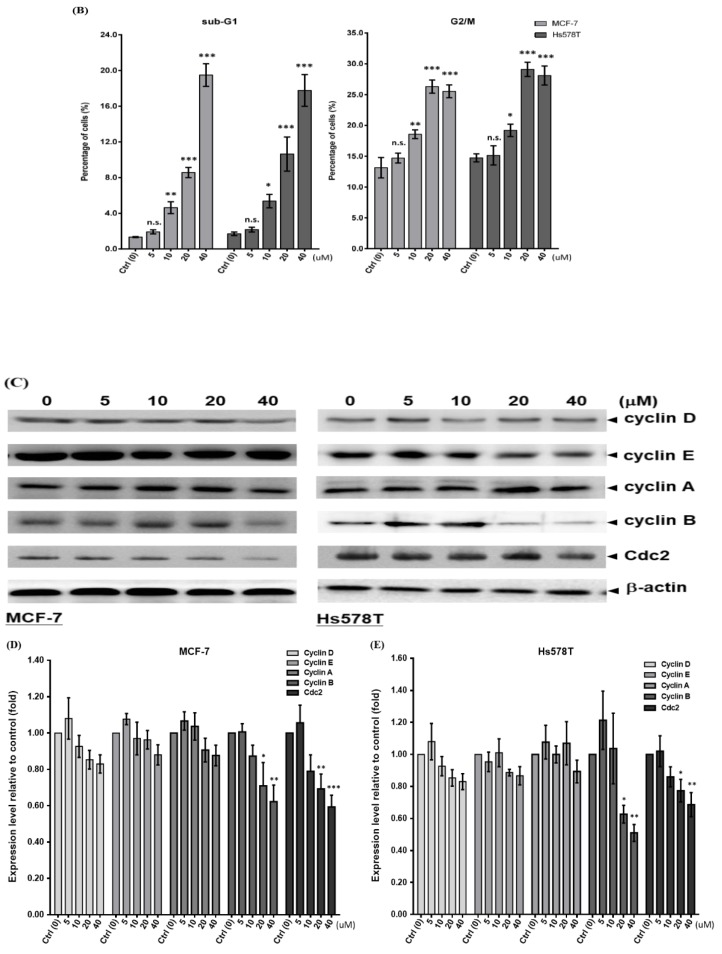
Effect of diosgenin on the induction of G2/M phase arrest in breast cancer cells. (**A**) Distribution of MCF-7 and Hs578T cells across each phase of the cell cycle as determined by flow cytometric analysis of the percentages of cells with specific DNA contents determined by propidium iodide staining. (**B**) MCF-7 and Hs578T cells were cultured in the absence or presence of the indicated concentrations of diosgenin for 48 h. Percentages of cells at different stages of the cell cycle are shown as means ± SD from three independent experiments. * *p* < 0.05, ** *p* < 0.01, and *** *p <* 0.001 vs. control (Ctrl, 0 µM) cells. (**C**) Diosgenin induces G2/M phase arrest in MCF-7 and Hs578T cells after diosgenin treatment (0–40 µM) for 48 h. (**D**,**E**) Protein levels were determined by Western blot analyses using specific antibodies to the indicated cell cyclins. β-actin was used to normalize the amount of protein in each lane. Data are presented as the mean ± SD from three independent experiments. * *p* < 0.05, ** *p* < 0.01, and *** *p* < 0.001.

Diosgenin treatment inhibits the proliferation of various human solid tumors [17,20,21,22,23]. In the current study, the cell cycle distribution of diosgenin-treated breast cancer cell lines was determined. As shown in Figure 2A, diosgenin induced concentration-dependent increases in the percentages of MCF-7 and Hs578T cells in the G2/M and subG1 phases using propidium iodide staining of the DNA content and flow cytometric analysis. Diosgenin (10 µM for 48 h) caused a significant proportion of breast cancer cells to enter the G2/M phase. In addition, there was a significant increase in cells in the sub-G1 phase at the same concentration and time of diosgenin treatment (*p* < 0.05) (Figure 2B).

Additionally, we examined the mechanism by which diosgenin caused G2/M phase arrest by measuring the expression of cell cyclins A, B, D, E, and Cdc2 with respect to p53-responsiveness (MCF-7 cells) and p53-non-responsiveness (Hs578T cells). As expected, the Western blot results (Figure 2C–E) indicated that decreased levels of cyclin B and Cdc2 in diosgenin-treated MCF-7 and Hs578T cells caused an increase in G2/M phase cell population.

### 2.3. Diosgenin Primarily Activates Chk1-Mediated Growth Arrest of Breast Cancer Cells

**Figure 3:**
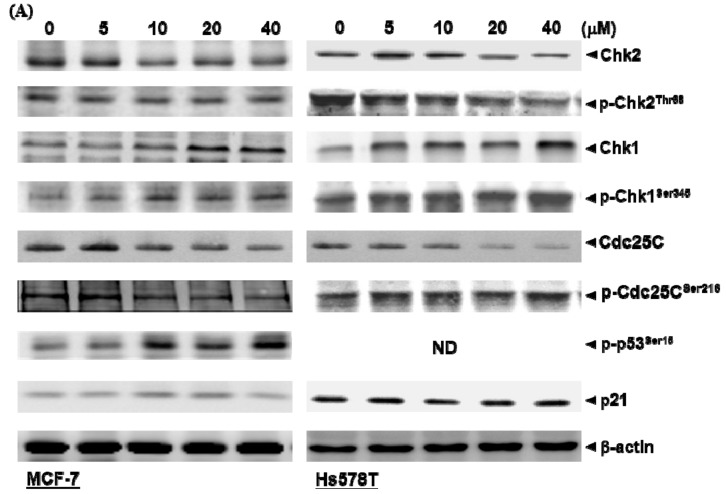

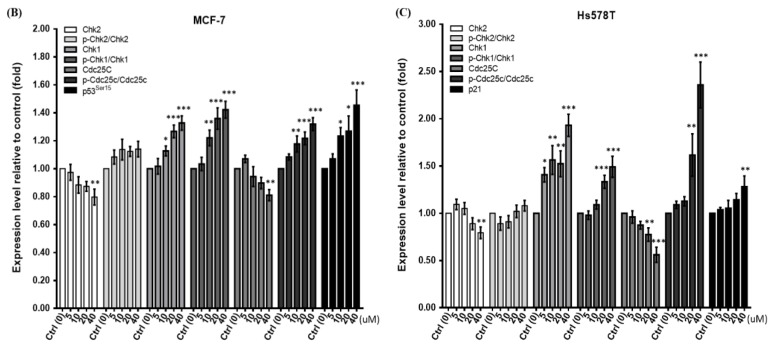
Diosgenin activates checkpoint proteins. (**A**) Incubation of MCF-7 (left panel) and Hs578T (right panel) cells with diosgenin increases expression of checkpoint kinase 1 (Chk1) and phosphorylated (activated) forms of Chk1 (p-Chk1^Ser345^), p-p53^Ser15^, p21, and p-Cdc25C^Ser216^. Densitometric analyses for (**B**) MCF-7 and (**C**) Hs578T cells were performed using Digital Protein DNA Imagineware. β-actin was used to normalize the amount of protein in each lane. Data are shown as means ± SD for three independent experiments. * *p* < 0.05, ** *p* < 0.01, and *** *p* < 0.001 vs. control (Ctrl, 0 µM) group.

Because the inhibition of cell cycle progression is associated with Chk phosphorylation, we explored the mechanism underlying p-Chk-induced Cdc25c phosphorylation, leading to G2/M arrest of diosgenin-treated cells. We observed significantly increased ratios of p-Chk1^Ser345^/Chk1 and p-Cdc25C^Ser216^/Cdc25C upon treatment with increasing concentrations of diosgenin for 48 h (Figure 3B,C). This indicated that Chk1-dependent phosphorylation (p-Chk1^Ser345^) of Cdc25C^Ser216^ is required, which, in turn, acts to sustain G2/M arrest in both breast cancer cell lines. Notably, diosgenin-induced activation of p-Chk1^Ser345^ markedly increased the phosphorylation level of p53 at Ser15 in MCF-7 cells treated with diosgenin above 10 µM for 48 h. Further, increased p21 protein was observed in diosgenin-treated Hs578T cells in which the p53 gene is inactive [24]. This indicates that the induction of p21 causing G2/M phase arrest in diosgenin-treated Hs578T cells is p53-independent (Figure 3A). In addition, we demonstrated that the inactivation of Cdc25c by diosgenin to cause G2/M phase arrest occurred in a predominately Chk1-dependent manner and was due to neither Chk2 nor p-Chk2^Thr68^, which were not significantly increased in long-term cultures with 40 µM diosgenin (Figure 3B,C). Taken together, these data indicate that diosgenin can specifically augment the expression levels of p-Chk1^Ser345^ accompanied with Cdc25C^Ser216^ phosphorylation that results in an alteration of G2/M-related proteins.

### 2.4. Diosgenin Induces Mitochondrial Dysfunction in Breast Cancer Lines

**Figure 4:**
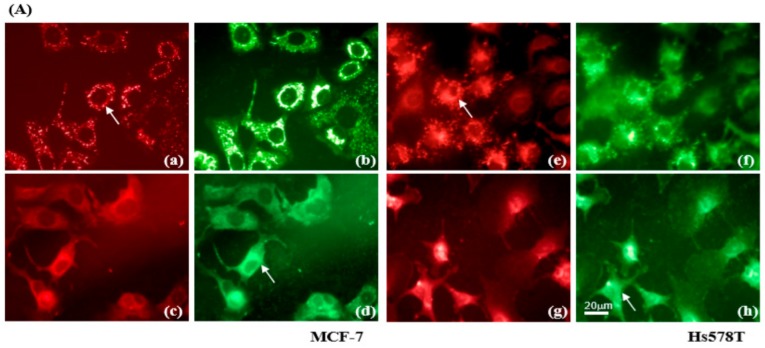

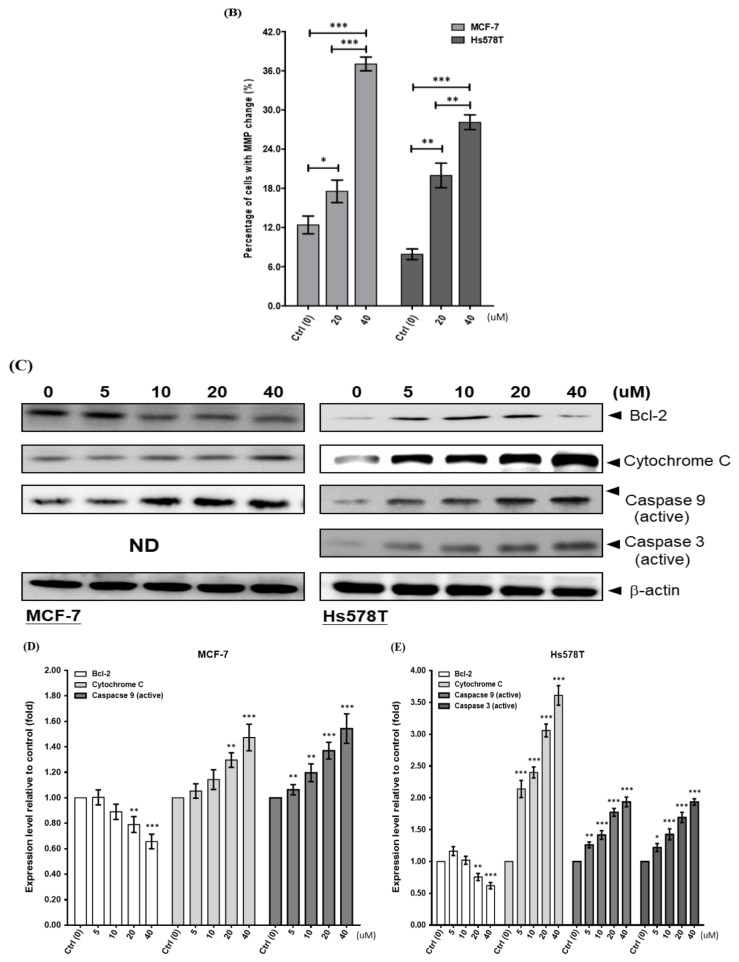
Diosgenin dissipates the mitochondrial membrane potential (∆Ψm) in breast cancer cells. (**A**) Images of JC-1 fluorescence were taken at 48 h. Red fluorescence indicates aggregates of JC-1 in intact mitochondria, whereas green fluorescence represents the JC-1 monomer in cytosol. MCF-7 and Hs578T cells treated with vehicle are shown in (**a**,**b**,**e**,**f**), respectively. MCF-7 and Hs578T cells treated with 20 µM diosgenin are shown in (**c**,**d**,**g**,**h**), respectively. Arrows indicate the translocation of JC-1 from mitochondria (**a**,**e**) to cytosol (**d**,**h**). (**B**) Quantitation of the flow cytometric analysis of diosgenin-treated MCF-7 (left panel) and Hs578T (right panel) cells showing loss of the ∆Ψm. Data are shown as means ± SD for three independent experiments. * *p* < 0.05, ** *p* < 0.01, and *** *p <* 0.001 vs. control (Ctrl, 0 µM) cells. (**C**) Breast cancer cells were treated with various concentrations of diosgenin for 48 h. Western blot analyses with antibodies to apoptosis-relevant proteins. β-actin was used as the loading control. (**D**,**E**) Protein levels were determined by Western blot analyses using specific antibodies to the indicated cell cyclins. β-actin was used to normalize the amount of protein in each lane. Data are shown as means ± SD for three independent experiments. * *p* < 0.05, ** *p* < 0.01, and *** *p* < 0.001 vs. control (Ctrl, 0 µM) group.

Mitochondria play a key role in the intrinsic pathway of apoptosis via loss of the mitochondrial membrane potential (∆Ψm), and mitochondria-mediated apoptosis can be initiated by activating the caspase signaling pathway. To investigate the molecular mechanism by which diosgenin-induced cell death occurred, we examined its effect on changes in the ΔΨm using flow cytometry to measure JC-1 fluorescence intensity. An appreciable increase in the percentage of green JC-1 fluorescence in diosgenin-treated MCF-7 and Hs578T cells occurred in a concentration-dependent manner (Figure 4A,B).

Loss of Bcl-2 protein results in cytochrome c release from mitochondria into cytosol [25]. Consistently, Bcl-2 was decreased in diosgenin-treated MCF-7 and Hs578T cells, triggering cleavage and activation of caspase-9 as compared to that in control cells (Figure 4C–E). Furthermore, an increased level of caspase-3 protein was evident in diosgenin-treated Hs578T cells (MCF-7 cells are deficient of caspase-3 [26]). These findings demonstrate that mitochondria-mediated apoptosis caused by diosgenin is involved in mitochondrial disruption, triggering caspase-dependent death of breast cancer cells.

## 3. Discussion

**Figure 5:**
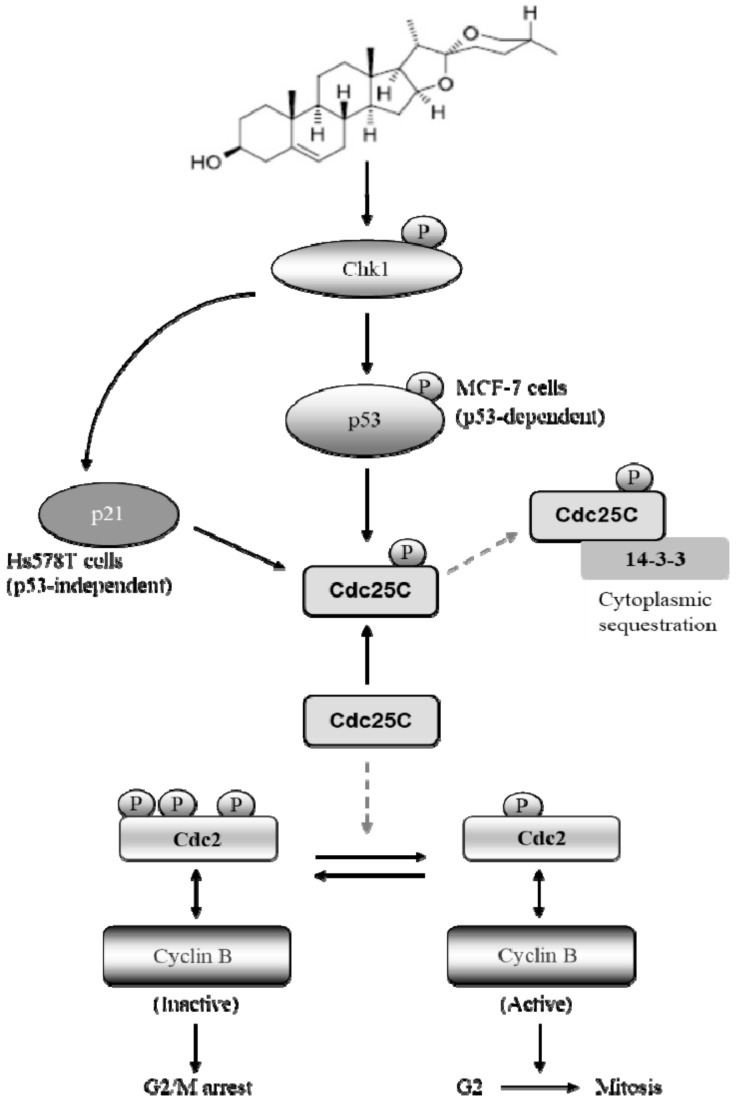
Model showing the proposed molecular mechanism of diosgenin-induced mitochondria-dependent apoptosis via checkpoint kinase activation in breast cancer cells. In this model, solid arrows show positive activation pathways determined in our studies. Gray arrows indicate functions reported previously [27]. The model suggests that diosgenin phosphorylates (activates) Chk1 at Ser345, leading to inactivation of the downstream target protein, phospho-Cdc25C^Ser216^. Alternatively, inhibition of Cdc2/cyclin B via phosphorylated Chk1-Cdc25C signaling results in G2/M phase arrest that can cause p53-independent p21 activation, as shown in Hs578T cells.

The results of the present study demonstrated that diosgenin exerted an anti-tumor activity on two human breast cancer cell lines, MCF-7 and Hs578T. The anti-proliferative activity of diosgenin involves the induction of G2/M phase arrest via modulating the Cdc25C-Cdc2-cyclin B pathway and mitochondria-mediated apoptosis in concentration- and time-dependent manners in both cell lines. To our knowledge, this is the first report describing the mechanism whereby the Chk1-activated p21-Cdc25C-Cdc2 signaling pathway plays a key role in regulating cell cycle progression in diosgenin-treated Hs578T breast cancer cells that are functionally deficient of p53 (Figure 5).

Laboratory research suggests that diosgenin, a steroidal saponin found in fenugreek seeds, may be an effective therapeutic agent against many types of human cancers, including breast cancer [28]. Emerging data have validated molecular targets and signaling pathways affected by diosgenin, and shown that it is very effective in suppressing cell growth in a variety of human cancers [13]. For example, diosgenin down-regulates NEDD4 expression to decrease growth and motility of prostate cancer cells [29]. Diosgenin exerts its tumor suppressive function to induce apoptosis by down-regulating MALAT1/STAT3 signaling in gefitinib-resistant non-small cell lung cancer [30]. In addition, diosgenin activates tumor cell autophagy and apoptosis by inhibiting the phosphorylation of phosphoinositide 3-kinase, Akt, and the mammalian target of rapamycin, thereby decreasing signaling through this pathway [31]. Furthermore, diosgenin can improve intestinal microbiota, which may increase immune functions to enhance the therapeutic efficacy of the checkpoint protein, PD-1, when treating melanoma [32]. Our results demonstrated that diosgenin induced time- and concentration-dependent inhibition of MCF-7 and Hs578T cell proliferation. Based on biochemical and flow cytometric analyses, we found that this inhibition involved arresting the cell cycle at the G2/M phase by negatively regulating Cdc2/cyclin B, even in the absence of p53 in Hs578T cells. This phenomenon is consistent with a previous report that diosgenin induces a significant cell cycle arrest at the G2/M phase, leading to p53-independent apoptosis of human leukemia cells [22].

Agents capable of deregulating expression of key components for maintenance of normal cell cycle progression in cancer cells may serve as candidates of prognostic assessments. Of the cell cycle regulators, Chk1 is situated at the crossroads of signaling pathways that function as ordinary regulators of cell growth and apoptosis. Chk1 has significant effects on the G2 and M phases in human cells during DNA damage repair. As a consequence, the antimitotic activities of alkaloids and soy consumption were investigated in terms of Chk1 phosphorylation [33,34]. Here, we demonstrated that diosgenin-induced G2/M arrest was mediated by phosphorylation of Chk1. Subsequently, phosphorylation of p53^Ser15^ and Cdc25C^Ser216^ was simultaneously enhanced in diosgenin-treated MCF-7 cells. Alternatively, the presence of p21 was required to suppress G2/M progression in diosgenin-treated p53-null Hs578T cells, and an increased level of p21 that correlated with apoptosis was observed concomitantly in lung cancer cells treated with fucosterol [29]. Recent studies have also ascertained that lauryl gallate induced growth inhibition of MDA-MB-231 breast cancer cells as well as garcinol-induced G1 arrest of H1299 lung cancer cells based on p53-null phenotypes [35,36]. Alternatively, a recent investigation of Chk1 has indicated that EGF stimulation induced phosphorylation of Chk1 at Ser280 residue (Chk1^Ser280^) via PI3K/Akt/p70S6K pathway, resulting in cell growth by activation of the downstream signal transduction [37]. Thus, to comprehensively understand the mechanism underlying Chk1-modulated downstream genes that interferes proliferation of breast cancer cell caused by diosgenin, consecutive studies to determine phosphorylation of Chk1 resided in different amino acid residue are required for cancer therapy.

Except cell cycle arrest, apoptosis may regulate cell proliferation. To explore the influence of mitochondrial apoptosis pathways of breast cancer cells, we utilized flow cytometric analysis with JC-1 to identify cells with a loss of the ∆Ψm. We also found increased levels of cytochrome c and activated caspase proteins in conjunction with a reduced level of Bcl-2 in both MCF-7 and Hs578T cells treated with diosgenin. In previous investigations, structural analoges of saponin were reported to dissipate the ∆Ψm, a prerequisite for tumor cell death [38,39]. Furthermore, activation of a series of caspase cascades after collapse of the ∆Ψm, followed by cytochrome c release, has been reported in cells sensitized by treatment with natural phytochemical compounds [33,40,41,42,43]. Intriguingly, we found a significant correlation between ∆Ψm collapse and deceased Bcl-2 following diosgenin treatment that led to an elevated level of activated caspases and promoted death of breast cancer cells (Figure 4). This is consistent with a previous report that decreased expression of Bcl-2 and reduction of the ∆Ψm occurred in diosgenin-treated HeLa cells [44].

In conclusion, the results of this study convincingly demonstrate that diosgenin treatment results in a decreased level of cyclin B1 and increased phosphorylation of Cdc25C^Ser216^ and Chk1^Ser345^, leading to cell cycle blockade at the G2/M phase, disruption of the ∆Ψm, and apoptotic cancer cell death. These findings provide molecular evidence suggesting that diosgenin could be a promising natural therapeutic agent and prototype for developing new antitumor strategies for treating human breast cancer.

## 4. Materials and Methods

### 4.1. Reagents

Diosgenin was purchased from Sigma-Aldrich (St. Louis, MO, USA). A stock solution of diosgenin was prepared in dimethyl sulfoxide and stored at −20 °C. For all assays, solutions were prepared freshly by dilution of the stock solution with phenol red-free Dulbecco’s modified Eagle’s medium (DMEM) (Life Technologies, Carlsbad, CA, USA). The same amount of vehicle (1% dimethyl sulfoxide) was added to control cells.

### 4.2. Cell Culture

Two breast cancer cell lines, MCF-7 (American Type Culture Collection; ATCC, Manassas, VA USA), HTB-22; caspase-3-deficient) and Hs578T (ATCC HTB-126; p53-deficient), and two non-tumorigenic human breast epithelial cell lines, HBL-100 (ATCC HTB-124) and H184B5F5/M10 (Bioresource Collection and Research Center, Hsinchu, Taiwan, BCRC-60197), were cultured in phenol red-free DMEM containing non-essential amino acids, 0.1 mM sodium pyruvate, 10% fetal bovine serum, 2 mM L-glutamine, 100 mg/mL streptomycin, and 100 units/mL penicillin (Biosource, Rockville, MD, USA). Cells were cultured at a constant temperature of 37 °C in a 5% carbon dioxide humidified atmosphere.

### 4.3. Cell Viability Assay

The effect of diosgenin on cell proliferation was determined using the [3-(4,5-dimethylthiazol-2-yl)-2,5-diphenyltetrazolium bromide] (MTT) assay. Cells were suspended at a final density of 5 × 10^4^ cells/mL/well using the Trypan blue examination method. After 24 h incubation, cells were harvested and treated with vehicle alone or the indicated concentrations of diosgenin for 24 and 48 h. After treatment, cells were rinsed with phosphate-buffered saline twice before incubation with MTT for an additional 4 h. The formazan generated, which is proportional to the number of viable cells, was dissolved in isopropanol and measured spectrophotometrically at 563 nm. The absorbance of control cells was defined as 100% viability.

### 4.4. Cell Cycle Analysis

To analyze the cell cycle distribution within a cell population, 1 × 10^6^ cells were plated in 60 mm dishes and treated with vehicle or various concentrations of diosgenin for the required times. At the end of the treatment, cells were harvested and resuspended in cold phosphate-buffered saline, fixed with 70% ethanol at −20 °C, then stained with 4 μg/mL propidium iodide (Sigma-Aldrich) solution containing 100 μg/mL of RNase and 1% Triton X-100. The fractions of cells in the subG1, G0/G1, S, and G2/M phases were analyzed with a FACSCalibur flow cytometer using the CellQuest Pro software (V5.2.2) (BD Biosciences, Franklin Lakes, NJ, USA).

### 4.5. Western Blot Analyses

The detailed procedure for Western blotting is described elsewhere [10]. Cells (1 × 10^6^) were cultured in triplicate in six-well plates containing various concentration of diosgenin for 48 h. Total cell extracts were prepared in ice-cold lysis buffer (0.5% NP-40, 50 mM Tris-HCl, 150 mM NaCl, 1 mM EDTA, and 10% glycerol, pH 7.5) containing protease inhibitors (1 μg/mL aprotinin, 0.5 μg/mL leupeptin, and 100 μg/mL of 4-(2-aminoethyl)-benzenesulfonyl fluoride). Equivalent amounts of proteins were resolved by 8–12% sodium dodecyl sulfate polyacrylamide gel electrophoresis and transferred onto a polyvinylidene difluoride membrane. Primary antibodies against human p21, p53, caspase-3, Chk1, cyclin D, Cdc25C, and Cdc2, or to rabbit cyclins (-A, -B, and -E), caspase-9, and Chk2 were supplied by Santa Cruz Biotechnology (Santa Cruz, CA, USA). Polyclonal antibodies to phosphorylated (p)-p53 (Ser^15^), p-Chk1 (Ser^345^), and p-Cdc25C (Ser^216^) were obtained from Cell Signaling Technology (Beverly, MA, USA) and to p-Chk2 (Thr^68^) from Oncogene Research Products (San Diego, CA, USA). The antibody against β-actin, used as the endogenous control to normalize the expression of proteins of interest, was obtained from Sigma-Aldrich. Immunoreactive bands were visualized by a chemiluminescence assay (Western blotting luminal reagent; Santa Cruz Biotechnology). Band intensities were quantified by densitometry (Digital Protein DNA Imagineware, Huntington Station, NY, USA).

### 4.6. Mitochondrial Membrane Potential Assay

To assess diosgenin-induced apoptosis relating to changes of the ∆Ψm, the fluorescent probe, 5,5′,6,6′-tetrachloro-1,1′,3,3′-tetraethylbenzimidazolyl-carbocyanine iodide (JC-1) (Molecular Probes, Eugene, OR, USA) was used. Monomeric JC-1, that emits green fluorescence, shifts reversibly to multimeric aggregates that emit red fluorescence when the ∆Ψm is high [45]. A total of 1 × 10^5^ cells were plated onto a dish containing a glass coverslip for 24 h. Then, attached cells on the glass coverslips were exposed to diosgenin for the required times. The coverslips were incubated with 25 μM JC-1 in loading buffer (150 mM NaCl, 5 mM KCl, 1 mM MgCl_2_, 2.2 mM CaCl_2_, 5 mM glucose, and 10 mM HEPES, pH 7.4) for 30 min at 37 °C, then mounted in a modified Cunningham chamber attached to the stage of an Eclipse TE2000-U microscope (Nikon Corporation, Tokyo, Japan), equipped with a Nikon 40× fluor objective. Images were collected with a cooled charge-coupled device camera (Coolsnap, Ropper Scientific, Vianen, The Netherlands) and processed with the Metafluor PC-software (Universal Imaging, West Chester, PA, USA). The numbers of cells with a fluorescence shift were measured using a FACScan flow cytometer (BD Biosciences) with detection at 590 nm (indicating intact mitochondria) to 525 nm (indicating leaky mitochondria).

### 4.7. Statistical Analyses

Data are presented as means ± standard deviation. Statistical significance of differences between experimental data grouped by one variable was assessed by the unpaired two-tailed Student’s *t*-test, or a one-way analysis of variance with the post-hoc Bonferroni-corrected *t* test, as appropriate. A value of *p* < 0.05 was considered to indicate statistical significance. All statistical analyses were performed using SPSS version 17.0 (IBM, Chicago, IL, USA).

## Abbreviations

Chk1	checkpoint kinase 1
Chk2	checkpoint kinase 2
CDK	cyclin-dependent kinase
CDKI	cyclin-dependent kinase inhibitor
Cdc25C	cell division cycle 25C
Cdc2	cell division cycle 2
MTT	3-(4,5-dimethylthiazol-2-yl)-2,5-diphenyltetrazolium bromide
∆Ψm	mitochondrial membrane potential
